# Lessons Learnt From Using the Machine Learning Random Forest Algorithm to Predict Virulence in *Streptococcus pyogenes*

**DOI:** 10.3389/fcimb.2021.809560

**Published:** 2021-12-24

**Authors:** Sean J. Buckley, Robert J. Harvey

**Affiliations:** ^1^ School of Health and Behavioural Sciences, University of the Sunshine Coast, Maroochydore DC, QLD, Australia; ^2^ Sunshine Coast Health Institute, Birtinya, QLD, Australia

**Keywords:** *Streptococcus pyogenes*, machine learning, random forest, virulence, phenotype metadata

## Abstract

Group A *Streptococcus* is a globally significant human pathogen. The extensive variability of the GAS genome, virulence phenotypes and clinical outcomes, render it an excellent candidate for the application of genotype-phenotype association studies in the era of whole-genome sequencing. We have catalogued the distribution and diversity of the transcription regulators of GAS, and employed phylogenetics, concordance metrics and machine learning (ML) to test for associations. In this review, we communicate the lessons learnt in the context of the recent bacteria genotype-phenotype association studies of others that have utilised both genome-wide association studies (GWAS) and ML. We envisage a promising future for the application GWAS in bacteria genotype-phenotype association studies and foresee the increasing use of ML. However, progress in this field is hindered by several outstanding bottlenecks. These include the shortcomings that are observed when GWAS techniques that have been fine-tuned on human genomes, are applied to bacterial genomes. Furthermore, there is a deficit of easy-to-use end-to-end workflows, and a lag in the collection of detailed phenotype and clinical genomic metadata. We propose a novel quality control protocol for the collection of high-quality GAS virulence phenotype coupled to clinical outcome data. Finally, we incorporate this protocol into a workflow for testing genotype-phenotype associations using ML and ‘linked’ patient-microbe genome sets that better represent the infection event.

## Introduction

*Streptococcus pyogenes* (group A *Streptococcus*: GAS) is a globally important, strictly human bacterial pathogen. Diseases caused by GAS are diverse in both severity and clinical outcome, and GAS infection impacts a range of tissues (Walker et al., 2014). The GAS genome comprises an arsenal of virulence factors, among the most preeminent of which is *emm*, which encodes the surface-exposed M protein (Walker et al., 2014). The nucleotide sequence of *emm* is the basis of the *emm* pattern genotyping system, and the strain-defining *emm* genotype (Bessen et al., 2018). The GAS genome is susceptible to recombination of endogenous and exogenous DNA (Feil et al., 2001). Collectively, this is important, because GAS kills over half a million people each year (Hand et al., 2020). Moreover, therapeutic options are a priority because no vaccine for GAS has been licenced (Vekemans et al., 2019), and hitherto GAS has shown exquisite susceptibility to penicillin, but recently several strains have shown resistance (Musser et al., 2020).

Comparative genomics, in the form of genome-wide association studies (GWAS) and machine learning (ML), has recently been successfully applied to large GAS genome datasets. Kachroo et al. have reported on a study of a population of the invasive *emm*28 GAS strain, which is over-represented in puerperal sepsis disease (Kachroo et al., 2019). The integrated design of this work encompassed genome, transcriptome, and virulence association testing using both ML and GWAS of 2101 genomes. The key finding of this study was that a single nucleotide deletion in the intergenic region (IGR) upstream of R28 was observed to significantly alter the global transcription profile and consequently the virulence of a subpopulation of isolates. In another study, Davies et al. used GWAS of 2083 GAS genomes in the successful assessment of vaccine candidate coverage encompassing 28 antigens (Davies et al., 2019).

Lees et al. conducted a GWAS of 5892 genomes of *Streptococcus pneumoniae*, a human pathogen capable of causing life-threatening invasive diseases (Lees et al., 2019). Importantly the genomes of the microbe and the patient human were simultaneously sampled and sequenced, constituting a set of genomes that is ‘linked’ and better represents the infection event. The proportional contribution of variation in the host and pathogen genomes to the infectious manifestation was attributed. This analysis demonstrated that the human genome accounted for about 50% of the susceptibility to meningitis, but only about 30% of the severity. By contrast, the bacterial genome explained 70% of the invasive potential, but had little effect on the severity. In other words, the serotypic strain of the bacteria was insufficient to explain invasive potential. Additionally, susceptibility to meningitis and invasive disease were observed to be associated with variation in a human gene (*CCDC33*) and nine pneumococcal genes (including some encoding adhesins, an endonuclease, and a putative carboxypeptidase). Studies of a similar design have used the nomenclature ‘joint’ (Lees et al., 2019), ‘co-genomic’ (Ebert and Fields, 2020), ‘plant-pathogen’ (Bartoli and Roux, 2017), and ‘genome-to-genome’ (human and HIV virus) (Bartha et al., 2013), highlighting the breadth of applicability of this burgeoning field.

We recently characterised the distribution and diversity of the nucleotide sequences of the two-component systems (TCSs) and stand-alone transcription regulators (TRs) in 944 GAS genomes, and then explored phenotype associations using phylogeny and concordance metrics (Buckley et al., 2018; Buckley et al., 2020). Subsequently, we applied the ML random forest (RF) algorithm to the allelic variation of the TRs to predict six metadata traits of the genomes (Buckley et al., 2021). These were *emm* type, *emm* subtype, country and tissue of the sample, propensity to cause invasive disease, and clinical outcome. We observed phylogeny-based association between the TR alleles and GAS strain (*emm* type), and were able to predict *emm* type using ML with 97% accuracy. However, no strong phylogeny-based associations were observed between the individual TR loci and infectious manifestation. Additionally, ML was used to predict *emm* subtype, country and invasiveness, but we were unable to usefully predict tissue tropism and clinical outcome. Significantly, three biological models were developed explaining rare recombination in the important genes of the *mga* regulon, that are detailed below.

The microbiology community is progressing towards accurate, real-time prediction of life-threatening infectious bacterial diseases and antibiotic resistance, using *in silico* techniques that exploit the increasing tractability and cost-efficiency, and decreasing lead-time of whole-genome sequencing (WGS). However, the field currently lacks easy-to-use, universally-applicable, end-to-end workflows (San et al., 2020). Furthermore, inconsistent with the increasing abundance of high-quality bacterial genomes, the field is hindered by a lack of *accompanying virulence phenotype metadata* that is of a standardised high quality. The aim of this review is to communicate the lessons learnt while applying ML to comparative genomics genotype-phenotype studies by contextualising our key outcomes within the framework of recent *Streptococcal* virulence studies.

## *In Silico* WGS-Derived Bacterial Comparative Genomics Techniques

In this era of next-generation sequencing, a multitude of *in silico* tools are being developed that leverage the high resolution of the abundance of WGS data that is being generated. Foundational to these tools are comparative genomic techniques that are informing our understanding of bacterial evolutionary history and epidemiology. A central hypothesis of comparative genomics is that variation in nucleotide sequences (effectively the genotype) correlates with the resultant phenotype (Chibucos et al., 2014). Two technologies at the forefront of comparative genomics are the GWAS and ML. Fundamental to these technologies are two contrasting strategies. While GWAS is an unbiased, whole-genome approach, ML strives to ‘pick winners’ in the form of candidate genes that are based on informed assumptions.

### Lesson 1: The Selection of Candidate Genes and the Pre-processing of Comparative Genomics Data

GAS TRs and TCSs are controllers of the initiation of transcription, in that they affect gene expression profiles and are key constituents of transcription regulatory networks. We hypothesised that variation in the alleles of these loci may correlate with virulence phenotypes. We curated a database of 944 GAS genomes with strain, geotemporal and phenotype metadata (where available), and compiled catalogues describing the allelic variability of 14 TCSs and 53 TRs. We also developed a novel allele-typing tool that was based on the ‘sort’ and ‘find unique’ algorithms. Consequently, we were able to identify many different recombination events, and that the most prevalent form of variation in these loci was the single-nucleotide polymorphism (SNP). Our curated database serves as a template for the use of GAS WGS-derived techniques in the selection of a candidate set of genes for comparative genomics studies.

## Phylogenetic Delineation

Phylogenetic delineation is central to the understanding of the evolutionary history and epidemiology of bacteria. In spite of the importance of *emm* as a GAS virulence factor, the quintessence of the *emm* type as the strain-defining, gold standard of GAS genotyping is under increasing scrutiny (Bessen et al., 2018; Davies et al., 2019). However, given that the vast majority of GAS epidemiological studies over the past century utilise the *emm*-based phylogenetic delineation, a vast *emm*-based knowledge prevails.

### Lesson 2: Bacterial Phylogenetic Delineation Needs a ‘WGS’ Redo

We observed a strain-dependent variability in the IGRs and coding sequences of GAS TCSs and TRs using phylogenetics and concordance, and proposed a set of core TRs as candidates for a novel GAS typing system. These subsequently informed the design of our ML workflow, in which we were able to predict the GAS strain (*emm* type) with 97% accuracy and establish that *mga*2 and *lrp* were the most mathematically powerful predictors of strain (Buckley et al., 2021). Overall this finding was important because it revealed a backward-compatibility between our TR-based typing system and the vast *emm*-based knowledge set.

Notably, *mga*2 and *lrp* are also biologically-significant TRs. *mga* is encoded adjacent to *emm*, regulates up to 10% of the genes of the GAS genome, and directly regulates the transcription of *emm*. *lrp* is encoded divergently adjacent to the streptokinase gene (*ska*) (Buckley et al., 2020), likely influencing its transcription. Where GAS streptokinase is capable of activating human plasminogen, which is a protein that dissolves blood clots, but not plasminogens of other mammalian species (Boyle and Lottenberg, 1997). Accordingly, although the molecular mechanism is to be determined, streptokinase is considered an important determinant in the human host specificity of GAS (Sun et al., 2004).

We discovered examples of rare recombination of the *mga* regulon including *mga*2-switching, *emm*-switching, and chimeric *emm*-*enn* events and were also able to develop evolutionary models to explain them (Buckley et al., 2020; Buckley et al., 2021). Furthermore, we identified the deletion of a transporter gene (*maeP*) that stands as a biomarker for the invasive *emm* subtype 89.0 (Buckley et al., 2018). Collectively, these findings were significant because it has been suggested (Lees et al., 2019) that an ability to detect rare genotype anomalies enhances the discovery of rare clinically-relevant phenotypes. We were also able to predict the country of origin using this approach, suggesting a geography-dependent evolution of GAS TRs (Buckley et al., 2021).

Throughout our studies, we have come to appreciate the virtues of the TR-based typing system over the *emm*-based systems for interpreting GAS phylogenetic delineation. The main advantages are: i) the absence of surface-exposure with the resulting lack of immunogenicity and positive selection pressure from host immunity; ii) multiple constituent genes that offer genomically-dispersed loci and an inherent redundancy, compared to the single ‘point of failure’ of the *emm* locus; iii) a range of recombinogenicities from which to select (Buckley et al., 2020); iv) a general absence of paralogues like *mrp* and *enn* encoded adjacent to *emm* that complicates the identification of *emm*; v) backwards compatibility to *emm*-based knowledge, and vi) the ability to detect rare *mga* anomalies, all whilst sharing the genotype-dependency and WGS-amenability of the *emm*-based systems. Placing this in context, we can see that the M protein was chosen as the original basis of phylogenetic delineation partly because it availed itself to the technology of the day, i.e. serotyping. We contend that the WGS era calls for the exploration of novel WGS-amenable typing systems, of which our TR-based system is one. Moreover, we contend that one typing system should not necessarily be assumed to be appropriate for both functions of phylogenetic delineation and interpretation of the biology of epidemiology.

## Bacterial Virulence

The global burden of infectious bacterial disease is significant. Whilst the terms ‘virulence’ and ‘pathogen’ have a foundational and pragmatic utility that endures, there is mounting evidence suggesting that these terms have fallen short as tools in the lofty ambition of fully elucidating microbial pathogenesis. Historical methods in this field used a framework that was pathogen-centric, reductionist, not dynamic, discretely binary in its classification, and limited by contemporary technology. Accordingly, there has been a shift of focus and an expansion of the scope of attention, so that in the WGS era we are advancing our approach to contextualise the host-microbe interactions into a dynamic continuum that accommodates a shift of an individual microbe from a harmful pathogen, to an opportunist, and even a commensal (Wiles and Guillemin, 2019). Where commensalism is a relationship between two organisms in which one benefits and the other derives neither benefit nor harm. All while accounting for the immunity and microbiome of the host, and abiotic environmental factors.

### Lesson 3: High-Quality Virulence Phenotype Metadata Is Crucial

By using pySEER to identify over-represented k-mers in the isolates displaying a propensity to cause invasive disease, Davies et al. had previously ascribed a binary phenotype classification to the genomes of our dataset (Davies et al., 2019). Where *k-mers* refers to the sets of complete and overlapping subsequences (k nucleotides in length) that are extractable from biological sequence (Ren et al., 2018). Using this information, ML and the variation in the TR alleles, we were able to predict invasiveness with high accuracy, and notably to 83% accuracy using only *mga*2 and *lrp*. The prediction power of this approach suggests tractable utility as a WGS-derived tool for pre-emptively inferring potentially life-threatening invasive GAS isolates in the clinical setting.

We were not able to usefully predict tissue preference using phylogenetics, concordance, or ML and the TR-based typing system. This was somewhat unexpected given that variation in the *mga* and *rofA*/*nra* TRs, and the *emm* pattern-associated landmarks of the *mga* regulon are known to correlate strongly with tissue tropism. Whilst we remain optimistic for the future application of comparative genomics, our methods and dataset were unable to elucidate the complexity of the GAS virulence phenotype. This was not unexpected given the complexity of GAS disease. However, the inability to predict these phenotypes is likely explained, at least in part, by the presence of the undefined or inaccurate values in the ‘tissue tropism’ and ‘clinical outcome’ fields of the input dataset. Recommendations to address these shortcoming are included below.

## Random Forest (RF) Machine Learning (ML) Algorithm

The RF ML algorithm is based on an ensemble of decision trees that are randomly generated from a set of input (or predictor) features, and the output of which is a majority vote of the trees that reduces the risk of an inaccurate prediction caused by any individual trees. In a supervised method, the rules for attaining the correct answer (label) are ‘learnt by example’, therein converting data into information. The RF is a robust and scalable method whose advantages include the ability to determine the importance of the predictor variable at predicting the correct answer. This is important because it allows for the elimination of statistically-dependent variables, reducing the dimensions of the input dataset and the computational resource usage. All of which is completed with highly interpretability.

### Lesson 4: Machine learning: Getting It Wrong Can Be So Right!

The RF algorithm was applied to the TR-based typing system to predict the selected strain-related, virulence phenotype, and geotemporal metadata. We were able to predict strain and geography with high accuracy, but were unable to predict virulence phenotype. By investigating the causes of inaccuracy in examples of where the predicted strain differed from the published strain, we were able to discover several rare anomalies in the *mga* regulon (Buckley et al., 2021). We identified a novel cell-wall spanning domain (SF5) which is described as a chimera of SF3 and SF1, that redefines the GAS *emm* pattern typing system. We also defined two categories of chimeric *emm*-*enn* events, where the resultant *emm* subtype is retained or changed, that we named ‘likewise’ and ‘contrariwise’, respectively. Finally, we proposed a model for the time-dependent excision of genes of the *mga* regulon.

Importantly, we have shown a utility for the RF algorithm in the interpretation of the phylogenetic delineation of GAS, while reducing the dimensions of the dataset and maintaining interpretability. Further, we have used ML to interpret the biology of GAS and propose new evolutionary models by establishing a workflow which serves as a template for testing hitherto untested GAS genomic traits. However, it should be noted that as part of dimension reduction the statistically dependent variables are excluded. Therefore, it is important to remember that whilst predictor variables correlate with the response variable (the answer), they are not necessarily causal. Conversely, an excluded variable could be causal or partially causal.

## Bacterial Genome-Wide Association Studies (GWAS)

GWAS is a comparative genomics technique that uses a suite of statistical models to test for associations or ‘statistical dependencies’ between variations in the DNA (genotype) of many genomes and the corresponding phenotype metadata, that may indicate causal relationships (Collins and Didelot, 2018). It is an unbiased methodology that can be performed on the whole-genome data without selecting candidate genes. While the traditional GWAS method that delineates phylogeny based on SNPs has yielded success in both human and bacterial genomes, an alternative method of increasing popularity uses k-mers.

Because they reproduce clonally, there are several considerations that hinder the application of GWAS techniques to bacteria that are not as relevant in human GWAS studies. The first of these is strong population structure or population stratification. Care must be taken to control for population structure to avoid identifying non-causal (spurious) relationships generated by: linkage disequilibrium with legitimately causal variants, environmental variables that are not controlled, and sampling errors induced by stratification (Earle et al., 2016). Current GWAS methods perform poorly in the presence of high linkage disequilibrium and population stratification in strongly clonal populations (Saber and Shapiro, 2020; Chen and Shapiro, 2021). This is important because it suggests that bacterial GWAS studies may improve with the application of novel methods that incorporate dimension reduction (Kwok et al., 2021) possibly using ML.

The recently published review by (Allen et al., 2021) articulates a utility for ML-based technologies in the synergistic complementation of the more established ‘statistical model’-based GWAS for the inference of bacterial virulence phenotypes. This study collated a list of considerations relevant to the design of ML and GWAS studies, and devised a general approach for identifying virulence genes using these comparative genomics techniques. The key steps included: pathogen collection, virulence measuring, WGS, identification of sequence variants, virulence association testing, and system confirmation or validation. Based on our findings, we envisage an increasing role of ML as an accompanying technology to GWAS comparative genomics, which is also generally agreed in the field (Lees et al., 2020; San et al., 2020; Allen et al., 2021).

## Discussion

### Quality Control Protocol for GAS Virulence Phenotype Data

We propose a quality control protocol for the collection of the virulence phenotype and clinical outcome data of GAS infection, with the benefit of qualifying a reportable metric. Our system allows for the continued analysis of existing data, while simultaneously incentivising the progressive production and consumption of higher- quality genomic metadata. At the time of sampling of the bacterial isolate for subsequent sequencing, we recommend that clinicians assign a classification for all of the following categories (from **Table 1** where appropriate): i) anatomical site of the sample; ii) tissue sampled; iii) clinical presentation; iv) end-point clinical outcome (where different from the presentation); and v) the classification of invasive, non-invasive, or non-suppurative sequela disease.

**Table 1:** Compilation of the expected classifications of tissues sampled, clinical presentation, and human patient risk factors in group A *Streptococcus* infection for use in a quality control protocol for the collation of high-quality virulence phenotype metadata.

Genomic metadata categories	Expected classifications
Tissue sampled^1^	Epithelial swab, blood, sputum, urine, saliva, synovial fluid, soft tissue, cerebrospinal fluid
Clinical presentation^1^	Throat carriage, scarlet fever, streptococcal toxic shock syndrome, type II necrotizing fasciitis, pharyngitis, superficial soft tissue infection, deep soft tissue infection, cellulitis, meningitis, pneumonia, bacteraemia, arthritis, puerperal sepsis, genital infection, iGAS, acute phlegmonous gastritis, rheumatic fever, rheumatic heart disease, post-streptococcal glomerulonephritis, paediatric autoimmune neuropsychiatric disorders associated with *Streptococcus* (PANDAS)
Human patient risk factors^2^	Blood antigen group (Vyas et al., 2020); serology that is indicative of prior GAS infection (anti-SLO or anti-DNase B antibodies); ethnicity; chronic liver disease; long-term alcohol abuse; homelessness, household crowding or relevant socioeconomic condition (Siemens and Lütticken, 2021); scabies as risk factor for pyoderma; immunity-suppressing pharmaceuticals; human immunodeficiency virus (HIV)/acquired immunodeficiency syndrome (AIDS); family history of GAS-related disease; and twin status (monozygotic or dizygotic).


^1^Expected classifications are adapted from the Davies GAS atlas (Davies et al., 2019), ^2^Compliance with human ethics standards is required.

**Figure 1:**
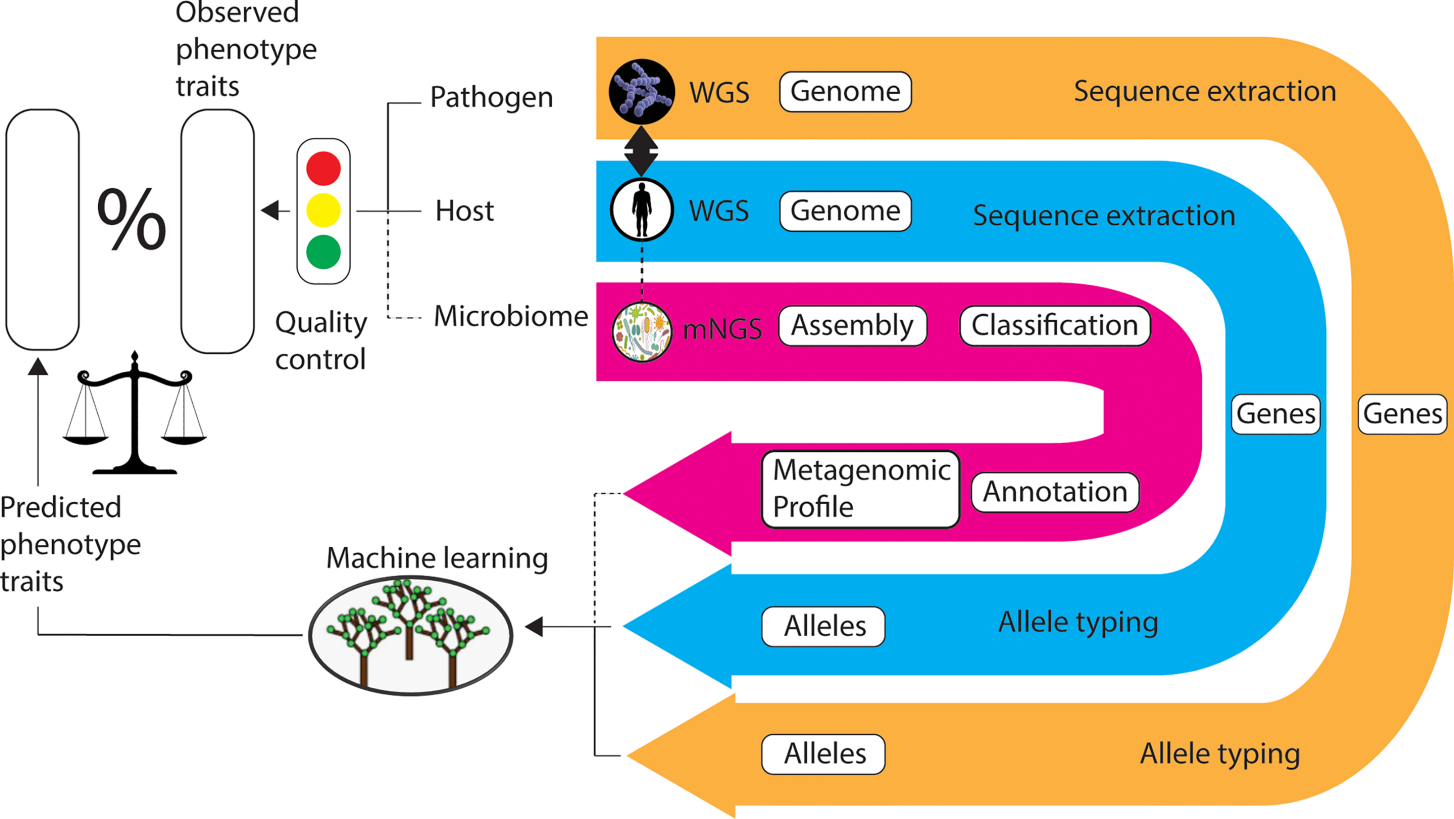
Workflow for the application of machine learning in comparative genomics genotype-phenotype association studies. ‘Linked’ samples of microbe, host, and microbiome (optional) are simultaneously collected and sequenced. Recommended virulence phenotype metadata is also collated and assessed for quality. The DNA sequences of target genes (for example, transcription regulators) are extracted and the alleles are typed. Machine learning algorithms are applied to the allele types (predictor variables) in the prediction of response variables (for example, invasive virulence phenotype). The machine learning models are validated by comparison of observed and predicted phenotype data. The most important predictor variables are selected as the basis of dimension reduction, as required. Legend: WGS, whole-genome sequencing; mNGS, metagenomic next generation sequencing.

By way of qualification of the quality control rating of the metadata collected, if no metadata is collected the rating is red. If each of i) to v) above has a non-null entry using **Table 1**, where applicable, the rating is green. All other circumstances are rated amber, using a simple traffic-light system that is readily interpreted (**Figure 1**). Moreover, where possible we urge the collection of human patient risk factor data. A non-comprehensive list of which is also included in **Table 1**.

### Applications

**Figure 1** depicts a workflow for the application of ML to ‘linked’ genome sets in the prediction of genotype-phenotype associations. We also envisage the introduction of microbiome metagenomics data in the future.

## Conclusions

The future application of comparative genomics in GAS genotype-virulence phenotype association studies is highly promising, and a key tool is the unbiased GWAS. Furthermore, we recommend the synergistic utility of ML with GWAS as a tool for dimension reduction and appraisal of ‘candidate genes’, all with high interpretability. We have suggested that the quality and abundance of bacterial phenotype data lags behind that of the accompanying genome data, and proposed a quality control protocol that incentivises the eventual improvement of the quality of GAS virulence phenotype data collection. Finally, we envisage the inevitable widespread use of ‘linked’ genome sets in eukaryotic host-microbe interaction studies and have developed a work flow for the application of ML to these sets.

## Author Contributions

SB and RH conceived the manuscript and selected the candidate journal. SB drafted and formatted the manuscript. RH revised the manuscript. SB is the intended first and corresponding author. All authors contributed to the article and approved the submitted version.

## Funding

SB and RH wish to acknowledge the generous support of the University of the Sunshine Coast.

## Conflict of Interest

The authors declare that the research was conducted in the absence of any commercial or financial relationships that could be construed as a potential conflict of interest.

## Publisher’s Note

All claims expressed in this article are solely those of the authors and do not necessarily represent those of their affiliated organizations, or those of the publisher, the editors and the reviewers. Any product that may be evaluated in this article, or claim that may be made by its manufacturer, is not guaranteed or endorsed by the publisher.
